# Limonin Exhibits Anti-Inflammatory Effects by Inhibiting mTORC1 and Mitochondrial Reactive Oxygen Species in Psoriatic-like Skin Inflammation

**DOI:** 10.3390/antiox13121541

**Published:** 2024-12-16

**Authors:** Seung Taek Lee, Jong Yeong Lee, Ha Eun Kim, Jun-Young Park, Jin Kyeong Choi

**Affiliations:** 1 Department of Immunology, Jeonbuk National University Medical School, Jeonju 54907, Republic of Korea; seungtaek98@naver.com (S.T.L.); shutp100@hanmail.net (J.Y.L.); gkdms21106@gmail.com (H.E.K.); 2 Department of Biochemistry, Chungbuk National University, Cheongju 28644, Republic of Korea; 3 Biomedical Research Institute of Jeonbuk National University Hospital, Institute for Medical Sciences, Jeonbuk National University, Jeonju 54907, Republic of Korea

**Keywords:** limonin, TPA, psoriasis, mitochondrial ROS, mTOR, AMPK

## Abstract

Psoriasis is a chronic inflammatory skin disorder characterized by abnormal immune responses and keratinocyte hyperproliferation. Limonin, a bioactive compound found in citrus fruits, has anti-inflammatory properties in various models; however, its effects on psoriasis are not fully understood. We investigated the therapeutic potential of limonin in a 12-O-tetradecanoylphorbol-13-acetate (TPA)-induced psoriasis mouse model. Mice were treated with TPA to induce psoriasis-like skin lesions, followed by intraperitoneal administration of limonin (200 or 400 μg/mouse) for six days. The results showed that limonin improved psoriasis-related symptoms in a psoriasis-like mouse model by suppressing the mRNA expression of pro-inflammatory cytokines and inflammation-related antimicrobial peptides and regulating the expansion of myeloid cells and T cells. Specifically, limonin reduced glucose uptake and oxidative phosphorylation to shift the metabolic program in the inflamed skin cells of psoriasis-like mice. Limonin activates AMPK and proteins related to mTOR inhibition, thereby suppressing the mTOR signaling pathway. It also inhibits mitochondrial mass and mitochondrial ROS production, thereby preventing the development of dysfunctional mitochondria in inflamed skin cells. Overall, limonin modulates key immune responses and metabolic pathways related to inflammation and mitochondrial health in psoriasis. Therefore, it is a promising natural candidate for the treatment of psoriasis and various inflammatory skin diseases.

## 1. Introduction

Psoriasis is a chronic inflammatory skin disease that affects millions of people worldwide. It is characterized by the rapid growth of skin cells, resulting in thick red patches covered with scales [1]. Psoriatic lesions often appear on the elbows, knees, scalp, and lower back; are often accompanied by intense itching and pain; and may even crack or bleed [1]. These visible symptoms have a substantial impact on the quality of life, and people with psoriasis are at high risk of depression [2]. Therefore, there is an urgent need for effective treatments to relieve symptoms and address the underlying causes of psoriasis.

The pathogenesis of psoriasis is driven by the abnormal proliferation of epidermal keratinocytes, inflammatory cytokines, and mediators secreted by immune-related cells, such as T and myeloid cells [3]. In particular, activated Th17 cells secrete key cytokines, such as IL-17, IL-22, and TNF-α, which play a key role in inducing keratinocyte over-proliferation, perpetuating the inflammatory response, and triggering chronic psoriatic inflammation [4,5,6]. Therapeutic approaches targeting Th17 have gained traction and showed rapid results in patients with psoriasis [7]. However, long-term use can have side effects, including increased susceptibility to bacterial and fungal infections [8].

A mechanistic target of rapamycin, mitochondrial function, and excessive reactive oxygen species (ROS) production (mTOR) plays a role in lesions [9,10,11]. The mTOR signaling pathway, which acts as a key regulator of inflammatory metabolism in psoriatic skin, induces cell growth and differentiation, contributing to the abnormal proliferation of keratinocytes [9]. In addition, these inflammatory metabolic signals cause skin cells to rely on glycolysis and mitochondrial oxidative phosphorylation (OXPHOS) to meet their energy needs, leading to excessive ROS production and mitochondrial dysfunction, resulting in cellular damage [12].

In this study, 12-O-tetradecanoylphorbol-13-acetate (TPA) was used to induce psoriasis-like inflammatory symptoms in mice [13]. TPA activates protein kinase C (PKC), leading to severe inflammation and rapid proliferation of keratinocytes, closely resembling the pathology of human psoriasis [14]. While imiquimod (IMQ), which activates Toll-like receptor 7 (TLR7), is also a commonly used model, the TPA model offers the advantage of more direct observation of the inflammatory response, as it relates to keratinocyte behavior [15].

Limonin, a member of the tetracyclic triterpenoid family of compounds found in citrus fruits, is a highly bioactive secondary metabolite found in plants that has garnered attention for its various health benefits, including anticancer, anti-inflammatory, and antioxidant effects [16,17,18,19,20]. Limonin attenuates LPS-induced lung injury by activating AMPK, a known mTOR regulator, and by inhibiting ROS production and abnormal mitochondrial growth in oocytes [21]. Furthermore, limonin was found to reduce atopic dermatitis symptoms by inhibiting MAPK signaling in TNF-α/IFN-γ-stimulated keratinocytes [22]. Limonin demonstrates high bioavailability and stable metabolic properties in humans, reaching peak plasma concentrations within approximately 6 h of ingestion, and shows no adverse effects even at a high dose of 2 g, making it a safe and promising therapeutic candidate [23]. However, its role in psoriasis-associated skin inflammation remains unclear.

In this study, we evaluated the potential of limonin as a natural candidate for psoriasis treatment. Limonin effectively suppresses inflammation by modulating changes in the metabolic profile of psoriatic skin cells in a TPA-induced psoriasis-like in vivo model and an in vitro keratinocyte model stimulated with IL-17.

## 2. Material and Methods

### 2.1. Chemicals

The chemical structure of limonin is shown in Figure S1. Limonin and rapamycin were purchased from Sigma-Aldrich (St. Louis, MO, USA) and SelleckChem (Houston, TX, USA), respectively.

### 2.2. Animals

Nine-week-old female C57BL/6J mice were purchased from NARA Biotech (Pyeongtaek, Korea) and housed in laminar flow cabinets. All animals were kept at 22 ± 2 °C and 55% ± 5% relative humidity and underwent a 12 h circadian cycle. All animal experiments shown in this study were conducted with the approval of the Animal Protection and Utilization Committee of Jeonbuk National University (JBNU IRB NON2024-163-001).

### 2.3. In Vitro Analysis

The human keratinocyte cell line HaCaT was obtained from CLS Cell Lines Service (Eppelheim, Germany). The cells were cultured in Dulbecco’s Modified Eagle Medium (DMEM) supplemented with 10% fetal bovine serum (FBS) and antibiotics (100 U/mL penicillin G and 100 μg/mL streptomycin) at 37 °C in a 5% CO_2_ incubator. HaCaT cells were treated with limonin (20 μg/mL), IL-17A (200 ng/mL; PeproTech, Cranbury, NJ, USA), and/or rapamycin (0.1 μM) in cell culture medium for 24 h. After treatment, the cells were harvested, and mitochondrial ROS and the effects of limonin were analyzed using qPCR (2 × 10⁴ cells/well), Western blot (1 × 10⁵ cells/well), and flow cytometry (FACS, 1 × 10⁴ cells/well). For transfection, HaCaT cells were transfected with siRNA targeting AMPKα1/2 (Santa Cruz Biotechnology, Dallas, TX, USA) or control siRNA (Santa Cruz Biotechnology, Dallas, TX, USA). Briefly, cells were transfected using FuGENE^®^ (Madison, WI, USA) SI transfection reagent according to the manufacturer’s instructions and incubated for 83 h. Following transfection, cells were treated with IL-17A and limonin for the specified time points prior to analysis.

### 2.4. TPA-Induced Psoriasis Model

The TPA-induced psoriasis-like skin lesion model in mice was established following previously described [24,25,26]. TPA was diluted in a 7:1 ratio of acetone to dimethyl sulfoxide (DMSO) and applied daily to both ears of each mouse at a dose of 2.5 μg/mouse from day 0 to day 6. Twenty-five mice were divided in to five groups, each containing five mice: control, TPA-induced, TPA with 200 μg/mouse limonin, TPA with 400 μg/mouse limonin, and TPA with rapamycin. Limonin was diluted in phosphate-buffered saline (PBS) at concentrations of 0.8 mg/mL and 1.6 mg/mL, and 250 μL of the diluted limonin was intraperitoneally injected into the limonin-treated groups at doses of 200 μg/mouse and 400 μg/mouse, respectively, from day 1 to day 6. Ear thickness was monitored as a measure of inflammation using a dial thickness gauge (Mitutoyo Co., Tokyo, Japan), and the body weight of each mouse was recorded. After the final treatment, the mice were euthanized using CO_2_ and ear samples were collected for further analysis. Portions of the ear tissue were stored at −80 °C for later RNA and protein analysis, while other sections were fixed in 4% formaldehyde for histopathological examination. To isolate single cells from the ear tissue, the samples were incubated in a fresh enzyme solution (RPMI-1640 medium containing 0.4 mg/mL Liberase from Roche (Basel, Switzerland) and penicillin/streptomycin) at 37 °C for 1.5 h. The tissue was then dissociated, filtered through a 70 μm cell strainer, and analyzed using FACS.

### 2.5. Florescence-Activated Cell Sorting Analysis

On day 7, ear tissues from all mouse groups were collected, minced, and incubated in Liberase containing medium (Roche, Basel, Switzerland) for 1.5 h. The cells filtered through a 70 μm nylon cell strainer and centrifuged at 400× *g* for 8 min. Red blood cells were lysed using ACK buffer (Quality Biological, Gaithersburg, MD, USA) and the remaining cells were washed with PBS. Isolated cells were stimulated with phorbol-12-myristate-acetate, ionomycin, and a Golgi plug (BD Pharmingen, San Diego, CA, USA) for 4 h. A viability staining kit (Invitrogen, Carlsbad, CA, USA) was used to exclude dead cells. The following antibodies were used to identify T cells, myeloid cells, and cytokines: anti-mouse CD4 (clone RM4-5, BioLegend, San Diego, CA, USA), anti-mouse IFN-γ (clone XMG1.2, BioLegend, San Diego, CA, USA), anti-mouse Foxp3 (clone FJK-16s, Invitrogen, Carlsbad, CA, USA), anti-mouse IL-22 (clone IL22JOP, Invitrogen, Carlsbad, CA, USA), anti-mouse IL-17A (clone TC11-18H10, BD Pharmingen, San Diego, CA, USA), anti-mouse RORγt (clone AFKJS-9, Invitrogen, Carlsbad, CA, USA), anti-mouse CD11b (clone M1/70, BioLegend, San Diego, CA, USA), anti-mouse F4/80 (clone BM8, BioLegend, San Diego, CA, USA), anti-mouse Ly6C (clone AL-21, BD Pharmingen, San Diego, CA, USA), anti-mouse Ly6G (clone 1A8, BD Optibuild, San Diego, CA, USA), and anti-mouse CD163 (clone S150491, BioLegend, San Diego, CA, USA). Fluorescently labeled antibodies were used for staining, and a <0.5% isotype control was set as the gating threshold. Flow cytometry was performed using the Attune NxT Flow Cytometer (Invitrogen, Carlsbad, CA, USA).

### 2.6. Histological Analysis

Ear tissues from all the groups (n = 25) were fixed in 4% formaldehyde and embedded in paraffin. Tissue sections were cut to 5 μm thickness and stained with hematoxylin and eosin. Skin thickness was measured using five randomly selected fields per group at ×200 and ×400 magnification.

### 2.7. Quantitative Real-Time Polymerase Chain Reaction (qPCR)

To assess the mRNA expression levels in ear tissues, we conducted qPCR. Total RNA was isolated using RNAiso Plus (Takara Bio, Kusatsu, Shiga, Japan) following the manufacturer’s protocol. First-strand cDNA was synthesized using RevertAid™ First Strand cDNA Synthesis (Thermo Fisher Scientific, Waltham, MA, USA). The cDNA was then mixed with SYBR premix (NanoHelix, Daejeon, Republic of Korea) for amplification, and the mRNA levels were normalized and quantified using the StepOnePlus Real-Time PCR System (Applied Biosystems, Foster City, CA, USA).

### 2.8. Oxygen Consumption Rate

Mouse ear skin tissue cells were pre-cultured in Seahorse XF RPMI medium, which contained 10 mM XF glucose, 2 mM XF glutamine, and 1 mM XF pyruvate. The cells were seeded at a density of 5 × 10⁵ cells per well in poly-D-lysine-coated XFp cell culture miniplates. OCR measurements were performed using the XFp Cell Mito Stress Test Kit (Agilent, Santa Clara, CA, USA) according to the manufacturer’s protocol and analyzed with the Seahorse XFp Analyzer (Agilent, Santa Clara, CA, USA).

### 2.9. Glucose Uptake Assay

Mouse ear skin tissue cells were seeded in a 96-well plate and incubated with 2-[N-(7-nitrobenz-2-oxa-1,3-diazol-4-yl) amino]-2-deoxy-D-glucose (2-NBDG, 0.01 mg/mL; Thermo Fisher Scientific, Waltham, MA, USA) at 37 °C for 30 min. After incubation, the cells were washed twice with PBS and stained with Fixable Viability Dye at 4° C for 15 min to selectively detect viable cells. The samples were then analyzed using an Attune NxT Flow Cytometer.

### 2.10. Mitochondrial ROS Rate

Mouse ear skin tissue cells and HaCaT cells were seeded in a 96-well plate and stained with Fixable Viability Dye (Invitrogen, Waltham, MA, USA) at 4 °C for 15 min. Subsequently, the cells were washed and incubated with MitoTracker Green FM and MitoSOX (Invitrogen, Carlsbad, CA, USA) at 37°C for 1 h. After incubation, the cells were washed twice, and the samples were analyzed using an Attune NxT Flow Cytometer.

### 2.11. Western Blotting

On day 7, ear tissues from all mouse groups were homogenized, and protein extracts were prepared for Western blotting. Tissue Protein Extraction Reagent (T-PER), PhosSTOP, and EDTA-free protease inhibitors were added to 200 μL of each sample. Samples were homogenized and centrifuged at 10,000× *g* for 5 min and the supernatant was collected. Protein extracts (20 μg per lane) were separated on a 6–12% gradient sodium dodecyl sulfate–polyacrylamide gel and transferred to a 0.45 μm nitrocellulose membrane (Bio-Rad, Hercules, CA, USA). The membranes were blocked with 5% bovine serum albumin in Tris-buffered saline with Tween 20 for 1 h at 37 °C, followed by overnight incubation at 4 °C with primary antibodies targeting phosphorylated and total forms of ribosomal protein S6 kinase (p70S6K), Raptor, eukaryotic translation initiation factor 4E-binding protein 1 (4E-BP1), AMP-activated protein kinase alpha (AMPKα), mTOR, and β-actin (Cell Signaling Technology, Danvers, MA, USA). After washing, membranes were incubated with secondary antibodies for 1 h at room temperature. Protein bands were detected using SuperSignal West Pico PLUS (Thermo Fisher Scientific, Waltham, MA, USA).

### 2.12. Statistical Analysis

Statistical analyses were performed using GraphPad Prism 9 software. The normality of the distribution was assessed using the Shapiro–Wilk normality test. One-way analysis of variance (ANOVA) or *t*-tests were used, followed by the Holm–Šídák post-hoc test. Statistical significance was considered at *p* < 0.05, and data are presented as mean ± standard error of the mean (SEM).

## 3. Results

### 3.1. Limonin Improves Symptoms and Suppresses Amplified Psoriasis-Related Genes in TPA-Induced Psoriasis Mouse

**Figure 1:**
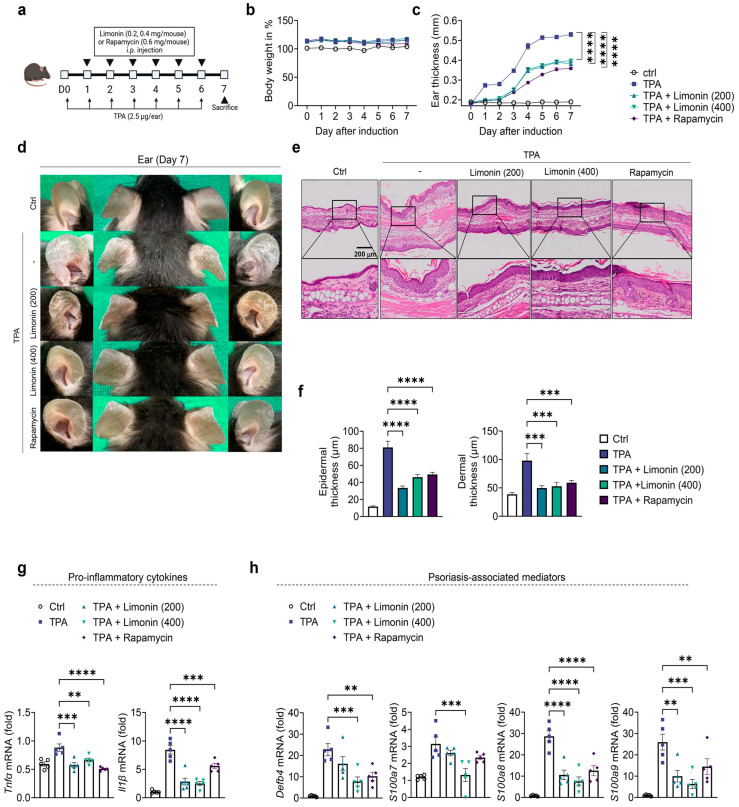
Limonin alleviates symptoms of TPA induced psoriasis-like mouse model and inhibits amplification of psoriasis-associated genes. (**a**) Experimental design for the induction of TPA-induced psoriasis-like mouse model. The mice (n = 4–6/group) were divided into five groups. (**b**) Mouse body weight change calculated as a percentage of the initial weight. (**c**) Ear thickness was measured 24 h after TPA application using a dial thickness gauge. (**d**) Representative photographs of mouse ears from each treatment group on day 7. (**e**) Representative photomicrographs of ear sections stained with hematoxylin and eosin (H&E) (×200 magnification; scale bar = 200 µm). (**f**) Epidermal and dermal thicknesses were measured using microphotographs of H&E-stained ear tissues. (**g**,**h**) Gene expression of pro-inflammatory cytokines (*Tnfα* and *Il1β*), and psoriasis-related antibacterial peptides (*Defb4*, *LCN*, *S100a7*, *S100a8*, and *S100a9*) in the ear skin of TPA and limonin or rapamycin-treated psoriasis-like mice; the ear skin was excised on day 7. Gene expression was analyzed using qPCR. The gene expression levels were normalized to that of β-actin. All data are presented as the mean ± SEM of four independent experiments. Values were analyzed by Holm–Šídák post-hoc test. ** *p* < 0.01, *** *p* < 0.001, **** *p* < 0.0001 vs. TPA-induced group. TPA: 12-O-tetradecanoylphorbol-13-acetate; H&E: hematoxylin and eosin; SEM: standard error of the mean; *Defb4*: defensin β 4; *S100a7*: S100 calcium-binding protein A7; *S100a8*: S100 calcium-binding protein A8; *S100a9*: S100 calcium-binding protein A9.

A TPA-induced psoriasis-like mouse model showed red scaly plaques accompanied by increased skin thickness. We used this TPA-induced mouse model to evaluate whether limonin alleviates psoriasis symptoms. As described in Figure 1a, limonin was administered by daily intraperitoneal injection at a dose of 200 or 400 μg per mouse for 6 days, and rapamycin was used as a positive control to investigate the effect of limonin on mTOR signaling. Treatment with limonin and rapamycin did not affect the body weight of the mice (Figure 1b). Ear thickness measurements showed that limonin reduced ear thickness at both low and high doses compared with that in the TPA-induced group (Figure 1c). The weights of the spleen and auricular lymph nodes were significantly lower in the limonin-treated group than those in the control group (Figure S2). Morphological and histopathological analyses also confirmed that limonin improved psoriasis symptoms compared with those in the untreated group (Figure 1d). Histopathological analysis showed that limonin significantly reduced TPA-induced increases in epidermal and dermal thickness at both low and high doses, similar to the effect of rapamycin treatment (Figure 1f). Furthermore, quantitative analysis of the expression of inflammatory cytokines and psoriasis-related mediators showed that limonin significantly inhibited the expression of inflammatory cytokines, such as *Tnfα* and *Il1β* (Figure 1g) and psoriasis-related mediators, such as *Defb4*, *S100a7*, *S100a8*, and *S100a9* compared with those in the TPA-induced group (Figure 1h).

### 3.2. Limonin Suppresses the Expansion of Inflammatory Myeloid Cells in the Skin of TPA-Induced Psoriasis Mouse Model

**Figure 2:**
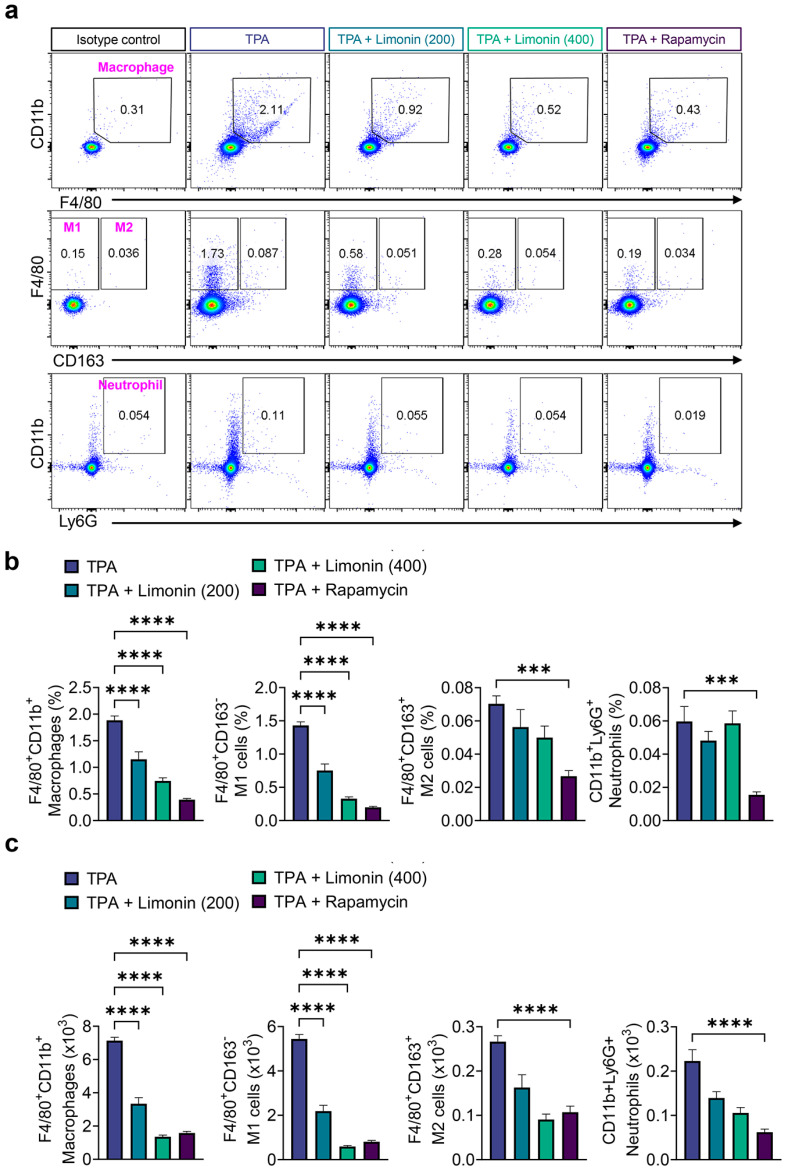
Limonin inhibits the innate immune cells in the skin of TPA-induced psoriasis mice. (**a**) Representative flow cytometry plots showing the percentages of Ly6G^+^CD11b^+^ (neutrophils), F4/80^+^CD11b^+^ (macrophages), F4/80^+^CD163^-^ (M1 macrophages), and F4/80^+^CD163^+^ (M2 macrophages) in skin cells. (**b**) Bar charts showing the percentages (upper panel) and (**c**) the numbers (lower panel) of Ly6G^+^CD11b^+^ (neutrophils), F4/80^+^CD11b^+^ (macrophages), F4/80^+^CD163^-^ (M1 macrophages), and F4/80^+^CD163^+^ (M2 macrophages) in skin cells. All data are presented as the mean ± SEM of four independent experiments. Values were analyzed by Holm–Šídák post-hoc test. *** *p* < 0.001, and **** *p* < 0.0001 vs. TPA-induced group. TPA: 12-O-tetradecanoylphorbol-13-acetate; Ly6G: lymphocyte antigen 6 complex, locus G.

Key players in initiating the inflammatory response in psoriasis are myeloid innate immune cells, such as macrophages, which are present in high density in the skin, contribute to the pathogenesis of psoriasis, and influence the activity of other immune cells [27]. Therefore, we investigated the effect of limonin on myeloid cells in the skin of TPA-induced psoriasis-like mice using flow cytometry. Limonin significantly suppressed the TPA-induced increase in macrophage (F4/80^+^CD11b^+^) expansion, similar to rapamycin. Furthermore, limonin significantly inhibited the pro-inflammatory M1 (F4/80^+^CD163^-^) phenotype of macrophages, but did not induce a switch to the anti-inflammatory M2 (F4/80^+^CD163^+^) phenotype, and did not suppress the increase in neutrophils (Ly6G^+^CD11b^+^) (Figure 2a–c).

### 3.3. Limonin Inhibits the Expansion of Th1/Th17 Cells and Induces the Production of Regulatory T Cells in TPA-Induced Psoriasis Mice

**Figure 3:**
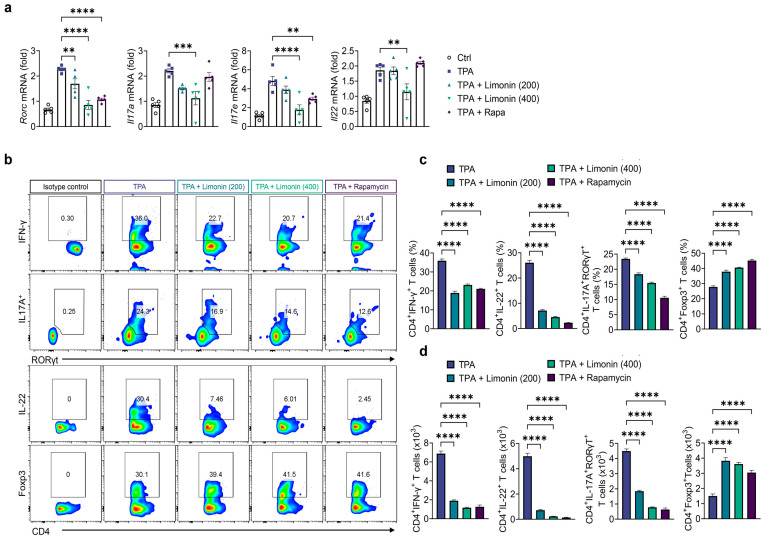
Limonin regulates T cell immune responses in the skin of TPA-induced psoriasis mice. (**a**) Gene expression of Th17-assciated genes (*Rorc*, *Il17a*, *Il17e*, *Il17f*, and *Il22*) in ear tissue of TPA and limonin or rapamycin-treated psoriasis-like mice, the back skin were excised on day 7. Gene expression was analyzed using qPCR. The gene expression levels were normalized to that of β-actin. (**b**–**d**) Representative flow cytometry plots and bar charts showing the percentage and numbers of CD4^+^IFN-γ^+^ (Th1 cells), CD4^+^RORγt^+^IL-17A^+^ (Th17 cells), CD4^+^IL-22^+^ (Th17 cells), and CD4^+^Foxp3^+^ (Treg cells) in the ear skin cells (*n* = 5/group). All data are presented as mean ± SEM of two independent experiments. Statistical significance was determined using the Holm–Šídák post hoc test. ** *p* < 0.01, *** *p* < 0.001, and **** *p* < 0.0001 vs. TPA-induced group. TPA: 12-O-tetradecanoylphorbol-13-acetate; Rorc: retinoic acid-related orphan receptor gamma c; IFN-γ: interferon gamma; IL-17A: interleukin-17A; IL-22: interleukin-22; Foxp3: forkhead box P3; Th1: T helper 1; Th17: T helper 17.

To investigate the effects of limonin on T cell-driven inflammation in a mouse model of TPA-induced psoriasis, we assessed its effects on T cell subpopulations and cytokine expression using qPCR and flow cytometry. Mice with TPA-induced psoriasis showed increased gene expression of both the Th17 transcription factor, RORC, and Th17-produced cytokines, Il17a, Il17e, Il17f, and Il22, in the ear tissue compared with those in normal mice. Additionally, limonin significantly suppressed Th17-related gene expression (Figure 3a). Furthermore, we isolated single cells from the skin of all mouse groups and examined the suppressive effects of limonin on Th1 and Th17 immune responses using intracellular cytokine staining. TPA-induced expansion of Th1 (CD4^+^IFN-γ^+^) and Th17 (CD4^+^IL-17A^+^RORγt^+^ and CD4^+^IL-22^+^) cells was significantly reduced in skin cells from TPA-induced mice treated with limonin and rapamycin, while simultaneously upregulating regulatory T cells (Treg, CD4^+^Foxp3^+^) (Figure 3b–d). These results suggest that limonin modulates T cell responses, thereby enhancing the anti-inflammatory effects on skin cells.

### 3.4. Limonin Reduces Mitochondrial ROS Production and Regulates Energy Metabolism in the Inflamed Skin of TPA-Induced Psoriasis Mice

In psoriasis, glucose uptake is increased in inflammatory skin cells and oxidative phosphorylation of mitochondria is activated to provide energy for inflammatory cell proliferation [28].

**Figure 4:**
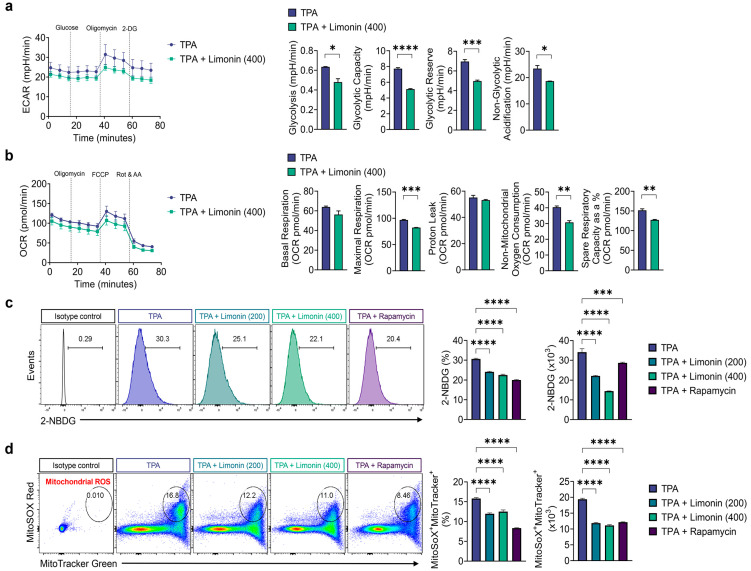
Limonin suppresses glycolysis, oxidative phosphorylation, and mitochondrial ROS in the skin of TPA-induced psoriasis mice. (**a**) Measurement of ECAR using a seahorse metabolic analyzer in ear skin cells from each group (left panel). Bar graphs show quantification of glycolysis, glycolytic capacity, glycolytic reserve, and non-glycolytic acidification (right panel). (**b**) Measurement of OCR using a Seahorse metabolic analyzer in ear tissue cells from each group (left panel). Bar graphs show quantification of basal respiration, maximal respiration, proton leak, non-mitochondrial oxygen consumption, and spare respiratory capacity (right panel). (**c**) Representative histograms of glucose uptake in ear tissue cells measured by using 2-NBDG (left panel) and bar graphs showing quantification of 2-NBDG in percentage and cell numbers (right panel). (**d**) Representative flow cytometry plots (left panel) showing mitochondrial ROS levels, as measured by MitoSOX Red, and mitochondrial mass, as measured by MitoTracker Green; bar graphs showing quantification of MitoSOX^+^ MitoTracker^+^ in percentage and cell numbers (right panel). All data are presented as mean ± SEM of four independent experiments. Statistical significance was determined using the Holm–Šídák post-hoc test. * *p* < 0.05, ** *p* < 0.01, *** *p* < 0.001, and **** *p* < 0.0001 vs. TPA-induced group. TPA: 12-O-tetradecanoylphorbol-13-acetate; OCR: oxygen consumption rate; ECAR: extracellular acidification rate; 2-NBDG: 2-[N-(7-Nitrobenz-2-oxa-1,3-diazol-4-yl) Amino]-2-Deoxyglucose.

Therefore, we analyzed the effects of limonin on the mitochondrial function and energy metabolism in a mouse model of TPA-induced psoriasis. First, we measured extracellular acidification rate (ECAR) and oxygen consumption rate (OCR) in skin cells from TPA-treated mice to identify changes in these processes and OXPHOS. In the skin cells of mice with TPA-induced psoriasis, glycolysis, glycolytic capacity, glycolytic reserve, and non-glycolytic acidification were significantly inhibited by limonin treatment (Figure 4a).

Furthermore, mitochondrial function profiles were examined using sequential treatment with oligomycin, FCCP, and rotenone for OCR measurements. Limonin significantly reduced maximal respiration, non-mitochondrial oxygen consumption, and spare respiratory capacity (Figure 4b). Glucose uptake was measured in TPA-induced psoriasis-like skin cells using the fluorescent glucose analog 2-NBDG and was significantly inhibited in the limonin- and rapamycin-treated groups, suggesting that this was related to the inhibition of glycolytic flux (Figure 4c). Furthermore, to determine whether inhibition of the mitochondrial metabolic profile by limonin prevents mitochondrial dysfunction, we measured the total mitochondrial content with MitoTracker Green and detected excess ROS production in mitochondria with MitoSOX Red. In the TPA alone-treated group, the fluorescence intensity of MitoTracker and MitoSOX increased, whereas the ROS produced by mitochondria was significantly inhibited in the limonin-treated group (Figure 4d).

### 3.5. Limonin Inhibits the mTOR Signaling Pathway by Inducing AMPK Activity in TPA-Induced Psoriasis Mice

**Figure 5:**
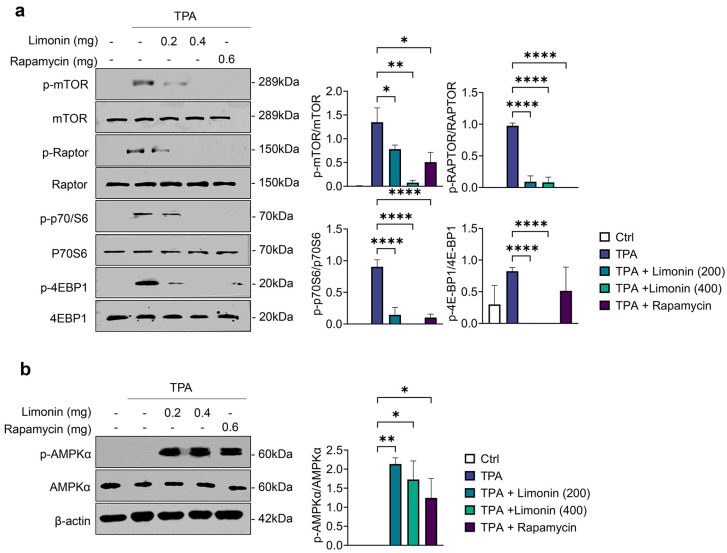
Limonin inhibits the mTOR signaling pathway by inducing AMPK activation. (**a**) Representative Western blot images of phosphorylated and total forms of key proteins involved in the mTOR signaling pathways (left panel) and bar graph of the relative intensities (right panel). (**b**) Representative Western blot images for p-AMPK, AMPK, and β-actin (left panel) and bar graph of the relative intensities (right panel). All data are presented as mean ± SEM of two independent experiments. Statistical significance was determined using the Holm–Šídák post-hoc test. * *p* < 0.05, ** *p* < 0.01, and **** *p* < 0.0001 vs. TPA-induced group. TPA: 12-O-tetradecanoylphorbol-13-acetate; mTOR: mechanistic target of rapamycin; RAPTOR: regulatory-associated protein of mTOR; p70S6K: p70 S6 kinase; 4E-BP1: eukaryotic translation initiation factor 4E-binding protein 1; AMPK: AMP-activated protein kinase.

Activation of mTOR regulates proteins and metabolism-related mechanisms required for cell proliferation and cytokine release and is largely influenced by energy levels [29]. In psoriatic skin, the upregulation of mTOR plays an important role in the proliferation of inflammatory cells and abnormal keratinocyte differentiation [29]. We investigated whether the anti-inflammatory effect of limonin regulates the expression of mTOR and its subcomplex, mTORC1, in TPA-induced psoriasis mice. Western blotting showed that the phosphorylation of the signal transduction pathway proteins Raptor, p70S6K, and 4EBP1 was activated in the ear tissues of TPA-induced psoriasis mice. However, this phosphorylation was significantly inhibited in limonin- and rapamycin-treated groups (Figure 5a). We also analyzed the expression of AMPK, a negative regulator of mTOR, to determine whether limonin regulates mTOR and mTORC1 activity. AMPK was significantly upregulated in limonin- and rapamycin-treated groups (Figure 5b). These results suggest that limonin can alleviate inflammation in TPA-induced psoriatic mouse skin by negatively regulating the mTOR signaling pathway through AMPK activity.

### 3.6. Limonin Regulates mTOR Signaling, Mitochondrial ROS, and Inflammatory Factors via AMPK in IL-17-Stimulated Keratinocytes

We demonstrated that limonin mediates mTOR inhibition via AMPK activation in an in vitro model of IL-17-stimulated psoriasis-like inflammation. To investigate this, we used an IL-17-induced inflammation model with human keratinocyte HaCaT cells, chosen as a stimulus because IL-17 induces inflammation and keratinocyte over-proliferation and plays an important role in psoriasis pathogenesis [4].

**Figure 6:**
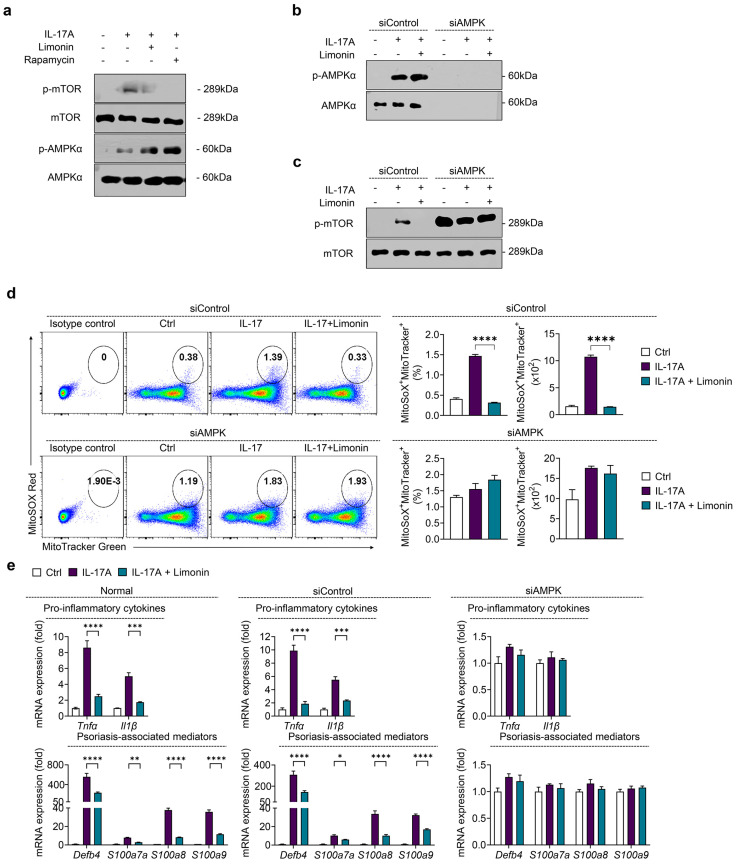
Limonin Inhibits mTOR Signaling and Mitochondrial ROS Production via AMPK Activation in IL-17-stimulated HaCaT cells. (**a**) Human keratinocytes HaCaT cells were stimulated with IL-17A (200 ng/mL) in the presence or absence of limonin (20 μg/mL) or Rapa (100 nM) for 24 h. Protein expression of mTOR and AMPKs was determined by Western blotting. (**b**–**e**) HaCaT cells were transfected with Control or AMPKs siRNA for 83 h and then stimulated with IL-17A (200 ng/mL) in the presence or absence of limonin (20 μg/mL) for 24 h (n = 3/group). (**c**) Cells were transfected either control or AMPK siRNA without stimulation and analyzed using Western blotting. (**d**) Representative flow cytometry plots (left panel) showing mitochondrial ROS levels, as measured by MitoSOX Red, and mitochondrial mass, as measured by MitoTracker Green; bar graphs showing quantification of MitoSOX^+^ MitoTracker^+^ in percentage and cell numbers (right panel). (**e**) Gene expression of pro-inflammatory cytokines (*Tnfα* and *Il1β*) and psoriasis-related antibacterial peptides (*Defb4*, *LCN*, *S100a7*, *S100a8*, and *S100a9*) were analyzed using qPCR. The gene expression levels were normalized to that of β-actin. All data are presented as mean ± SEM of independent experiments. Statistical significance was determined using the Holm–Šídák post-hoc test. * *p* < 0.05, ** *p* < 0.01, *** *p* < 0.001, and **** *p* < 0.0001 vs. IL-17-stimulated group. mTOR: mechanistic target of rapamycin; AMPK: AMP-activated protein kinase; ROS: reactive oxygen species; *Defb4*: defensin β 4; *S100a7*: S100 calcium-binding protein A7; *S100a8*: S100 calcium-binding protein A8; *S100a9*: S100 calcium-binding protein A9.

First, we determined a non-cytotoxic limonin concentration of 20 μg/ml using an MTT assay (Figure S3) and confirmed by Western blot that limonin treatment inhibited mTOR and strongly activated AMPK in the in vitro model (Figure 6a). Subsequently, siAMPK transfection was performed to elucidate the role of AMPK in mTOR signaling in IL-17-stimulated keratinocytes treated with limonin. siAMPK-transfected cells showed effective knockdown of AMPK (Figure 6b), and as expected, mTOR phosphorylation was reduced in the siControl group treated with IL-17 and limonin, but no inhibition of mTOR activation was observed in the siAMPK-transfected group (Figure 6c).

Furthermore, flow cytometry analysis utilizing MitoTracker and MitoSOX fluorescent staining to assess mitochondrial ROS production showed that limonin significantly reduced mitochondrial ROS levels in the siControl group compared to IL-17 alone, but this effect was not observed in the siAMPK group (Figure 6d). Similarly, qPCR analysis showed that RNA expression of psoriasis-related cytokines (Tnfα and Il1β) and psoriasis-related antibacterial peptides (Defb4, S100a7, S100a8, and S100a9) in IL-17-stimulated HaCaT cells was significantly suppressed by limonin in the siControl group, but this suppression was not observed in the siAMPK group (Figure 6e).

Collectively, these findings suggest that limonin plays a central role in inhibiting mTOR signaling through AMPK activation, regulating mitochondrial ROS production in IL-17-stimulated keratinocytes, and reducing the expression of psoriasis-related inflammatory factors.

## 4. Discussion

In this study, we validated the ability of limonin to ameliorate dry inflammation and metabolic dysregulation in a TPA-induced psoriasis-like mouse model and investigated its potential as a natural candidate for psoriasis treatment. Our findings suggest that limonin exerts anti-inflammatory effects on psoriatic skin lesions by restoring the altered metabolic program under psoriasis-like inflammatory conditions, downregulating the mTOR signaling pathway, and inhibiting mitochondrial ROS production.

Psoriasis is the most common chronic inflammatory skin disease and is initiated and maintained by abnormal epidermal keratinocyte proliferation and the interaction between innate and adaptive immune cells, including T cells, macrophages, and neutrophils [30,31]. Particularly, among activated T cells, Th1 cells produce IFN-γ, which activates macrophages, and Th17 cells release IL-17A, IL-17E, IL-17F, and IL-22, which play a role in the pathogenesis of psoriasis. Secreted IL-17 acts mainly directly on keratinocytes to stimulate the production of cytokines and antimicrobial peptides (Defb4, S100a7, S100a8, and S100a9). These molecules are increased in psoriatic lesions, attracting neutrophils, macrophages, and lymphocytes [32].

In this study, we showed that limonin was effective in treating psoriasis by alleviating the clinical manifestations of severe psoriasis in TPA-induced psoriasis-like mouse dermatitis and significantly suppressing the expression of antimicrobial peptides and cytokines associated with psoriasis. Limonin inhibits S100A8 and S100A9, which are biomarkers of psoriatic disease activity, suggesting that limonin may inhibit the excessive proliferation of keratinocytes that are strongly induced by IL-17 [33]. Furthermore, limonin significantly reduced the ratio of total macrophages to M1 macrophages, demonstrating that it can simultaneously control M1 polarization of skin macrophages and IFN-γ production by Th1 cells, inducing a positive feedback through the interaction between innate and adaptive immune cells.

Cytokines produced by Th17 cells play an important role in psoriatic inflammatory processes by regulating other stromal T cells, in addition to acting directly on keratinocytes. A recent study showed that excessive Th17 cell-produced cytokines can impair the immune tolerance of Tregs [34]. However, the results of this study suggest that limonin effectively modulates the excessive immune response in psoriasis by alleviating the inflammatory environment driven by Th17 cells in TPA-induced psoriatic skin inflammation, supporting the expansion of regulatory T cells.

Skin immune cells actively metabolize nutrients present in the microenvironment [35], which is particularly important in chronic inflammatory skin diseases, such as psoriasis. Psoriasis is characterized by the excessive proliferation of keratinocytes and expansion of inflammatory cells, implying increased intracellular nutrient requirements [35]. Glucose is the primary energy source for mammalian cells and drives glycolysis and the TCA circuit [35]. In human skin homeostasis and inflammation, metabolic reprogramming is induced by glycolysis and OXPHOS [35]. Here, we show that limonin controls glucose uptake and inhibits this process and OXPHOS in psoriasis-like inflamed mouse skin cells, the first results demonstrating not only its anti-inflammatory efficacy, but also its ability to reverse metabolic programs in an inflammatory environment.

Mitochondria have recently become a critical point in the immune system and can regulate inflammatory responses through the interplay between oxidative stress and metabolic reprogramming of cells [28]. Accumulating evidence suggests that mitochondrial function is impaired in skin diseases [35] owing to increased intracellular energy demands through inflammatory cell expansion [28]. Dysfunctional mitochondria exhibit increased mitochondrial biosynthetic mass and ROS production because of metabolic stress [28]. Therefore, controlling the ROS levels produced by dysfunctional mitochondria may represent a novel therapeutic target in a variety of skin diseases [28]. In the present study, limonin significantly suppressed mitochondria-produced ROS in a TPA-induced psoriasis model, suggesting a role of limonin in maintaining mitochondrial health during skin inflammation.

In psoriasis, mTOR plays an important role in the regulation of energy metabolism and is a key signaling pathway that regulates the activation and proliferation of immune cells and keratinocytes [35]. mTOR is composed of two protein complexes, mTORC1 and mTORC2, with the essential adaptor proteins, RAPTOR and RICTOR, respectively [12,35]. In psoriasis, exposure to IL-17 activates mTORC1, which contributes to the development of psoriasis by promoting Th17 cell differentiation and suppressing Treg cells through the phosphorylation of a subprotein called p70S6 and 4EBP [35]. mTORC1 activity is linked to glycolysis, oxidative stress, and mitochondrial dysfunction [36]. Our findings suggest that limonin plays an important role in attenuating the inflammatory response in psoriasis by inhibiting mTOR and mTORC1 activity. This suggests that in psoriatic dermatitis with an increased expansion of Th1/Th17 cells, limonin may contribute to maintaining the homeostasis of skin immune cells by regulating the mTORC1 pathway. In addition, limonin increased the activity of AMPK, a negative regulator of mTOR. These results suggest that limonin regulates the bioenergetic balance and hyperactivation of inflammatory cells in psoriasis by upregulating the AMPK pathway and inhibiting the mTOR signaling pathway. However, since psoriasis has a highly complex pathological mechanism, further research is needed to understand whether limonin also affects other metabolic signaling pathways.

## 5. Conclusions

Rapamycin is a well-established mTOR inhibitor that underscores the critical role of the mTOR signaling pathway in the pathogenesis of psoriasis. It offers promise as an anti-psoriatic therapeutic strategy through localized mTOR inhibition [37]. However, its systemic administration is often associated with limitations, including potential side effects and complications during long-term use [38].

In contrast, the present study provides preliminary preclinical evidence that limonin exhibits robust anti-inflammatory effects in psoriasis-like skin lesions. These results highlight the potential of limonin as a naturally occurring therapeutic candidate for skin diseases, such as psoriasis, offering a safer alternative to conventional treatments, including biologics or corticosteroids. Furthermore, limonin demonstrates additional therapeutic potential by modulating key metabolic mechanisms involved in skin immune diseases.

Compared to rapamycin, limonin showed comparable, and in some cases superior, efficacy in a psoriasis-like inflammation model. It not only suppressed inflammation and regulated immune responses effectively but also exhibited dual mechanisms by inhibiting mTOR signaling and activating the AMPK pathway. This dual action enables limonin to modulate both inflammatory and metabolic pathways, addressing the multifaceted pathogenesis of psoriasis. These findings suggest that limonin could overcome the limitations of current treatments, such as systemic side effects of mTOR inhibitors, and serve as a promising alternative therapeutic candidate for psoriasis and related inflammatory skin diseases.

## Data Availability

The data supporting the findings of this study are available from the corresponding author upon reasonable request.

## Supplementary Materials

The following supporting information can be downloaded at: https://www.mdpi.com/article/10.3390/antiox13121541/s1, Figure S1: Chemical structure of limonin; Figure S2: Representative photographs of mouse spleen and lymph node from each treatment group on day 7; Figure S3: Cell viability of human keratinocytes after limonin treatment.
