# Supplementary material for: Limonin Exhibits Anti-Inflammatory Effects by Inhibiting mTORC1 and Mitochondrial Reactive Oxygen Species in Psoriatic-like Skin Inflammation
Source: Antioxidants (Basel). 2024 Dec 16;13(12):1541. doi: 10.3390/antiox13121541 (PMC11727202; doi:10.3390/antiox13121541)
Supplement: Supplementary file 1 [file antioxidants-13-01541-s001.zip › antioxidants-3298074-supplementary.pdf]

Supplementary Information for

Limonin exhibits anti-inflammatory effects by inhibiting mTORC1 and mitochondrial reactive oxygen species in psoriatic-like skin inflammation

Seung Taek Lee, Jong Yeong Lee, Ha Eun Kim, Jun-Young Park, Jin Kyeong Choi

Corresponding author: Jun-Young Park and Jin Kyeong Choi

E-mail: jypark919@cbnu.ac.kr; jkchoi@jbnu.ac.kr

Files includes:

Methods

Figures S1, S2 and S3

Methods

MTT assay

Limonin (20 µg/mL) was added to the cell culture medium, and cell viability was assessed after 24 hours using 3-(4,5-dimethylthiazol-2-yl)-2,5-diphenyltetrazolium bromide (MTT) from Sigma-Aldrich (St. Louis, MO, USA). For this analysis, MTT (5 mg/mL) was added to each well, and the plate was incubated for 2 hours. Formazan crystals formed were dissolved with DMSO, and the absorbance of each sample was measured and expressed as a percentage relative to the control group.

Forward and reverse primer sequences for quantitative PCR analysis

Mouse

Gene	Forward primer (5'>3')	Reverse primer (5'>3')	Gene accession number
Tnfα	GGC AGG TCT ACT TTG GAG TCA TTG C	ACA TTC GAG GCT CCA GTG AAT TCG G	NC_000083.7
Il1β	CCA AAA GAT GAA GGG CTG CTT	TGC TGC TGC GAG ATT TGA AG	NC_000068.8
Defb4	CAG TCA TGA GGA TCC ATT ACC TT	AAT TTG GGT AAA GGC TGC AAT	NC_000074.7
S100a7	CCC TGC ACC AAG AGC AAC	GCACAGTTTTGTGGGG TTTT	NC_000069.7
S100a8	ATC ACC ATG CCC TCT ACA AGA ATG	GTC CAA TTC TCT GAA CAA GTT TTC G	NC_000069.7
S100a9	CAC CCT GAG CAA GAA GGA AT	TGT CAT TTA TGA GGG CTT CAT TT	NC_000069.7
Rorc	TGAGGCCATTCAG TATGTGG	CTTCCATTGCTCCTGCT TTC	NC_000069.7
Il17a	CTCAAAGCTCAGC GTGTCCAAACA	TATCAGGGTCTTCATTG CGGTGGA	NC_000067.7
Il17e	CCCCTGGAGATAT GAGTTGGAC	GTCTGTAGGCTGACGC AGTG	NC_000080.7
Il17f	CAGGAAGACAGC ACCATGAA	TCTTCTCCAACCTGAAG GAATTAG	NC_000067.7
Il22	AT GAG AGA GCG CTG CTA CCT GG	AAGGACGCCACCTCCT GCAT GT	NC_000076.7
β-actin	ACCCTAAGGCCAA CCGTGAA	ATGGCGTGAGGGAGA GCATAG	NC_000071.7

Human

Gene	Forward primer (5’>3’)	Reverse primer (5’>3’)	Gene accession number
Tnfα	GAGCTGAGAGATA ACCAGCTGGTG	CAGATAGATGGGCTCAT ACCAGGG	NC_000006.12
Il1β	GCTGATGGCCCTA AACAGATGAA	TGAAGCCCTTGCTGTAG TGGTG	NC_000002.12
Defb4	TGATGCCTCTTCCA GGTGTT	GCCTCCTCATGGCTTTT TGC	NC_000008.11
S100a7	ACGTGATGACAAG ATTGACAAGC	GCGAGGTAATTTGTGCC CTTT	NC_000001.11
S100a8	AGACCGAGACCGA GTGTCCTC	TGCCACGCCCATCTTTA T	NC_000001.11
β-actin	AGAGCTACGAGCT GCCTGAC	AGCACTGTGTTGGCGTA CAG	NC_000007.14

Figures S1

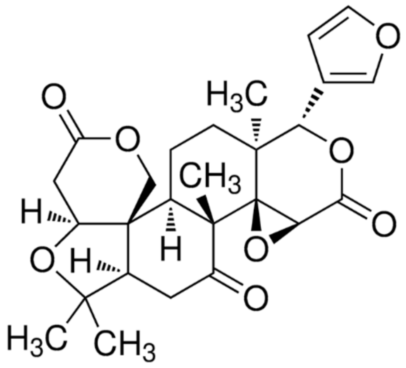

Figure S1: Chemical structure of limonin.

Figures S2

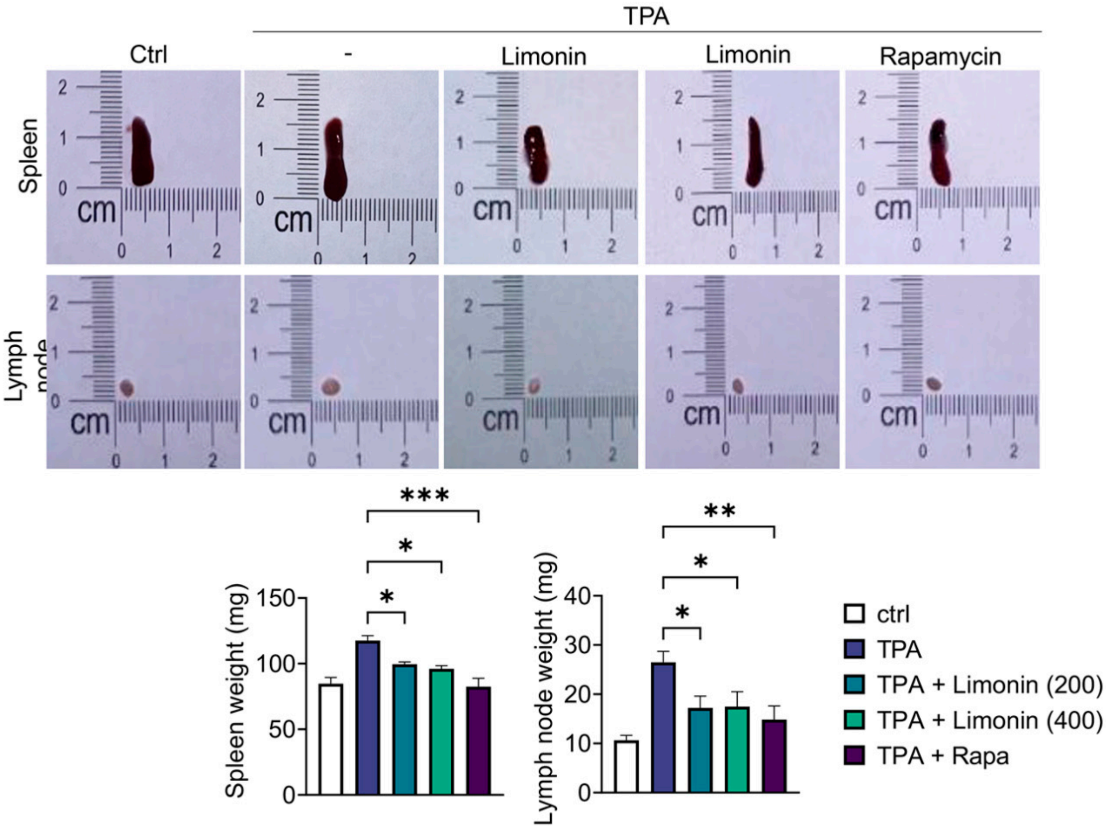

Figure S2: Representative photographs of mouse spleen and lymph node from each treatment group on day 7. TPA treatment induced visible gain of size, which were attenuated by limonin and rapamycin treatment. Quantification of spleen and lymph node weight (mg). Limonin treatment significantly reduced both spleen and lymph node weight compared to the TPA-induced group. All data are presented as mean ± SEM of two independent experiments. Statistical significance was determined using the Holm-Šídák post hoc test. *p < 0.05, **p < 0.01, and ***p < 0.001 vs. TPA-induced group. TPA, 12-O-tetradecanoylphorbol-13-acetate.

Figures S3

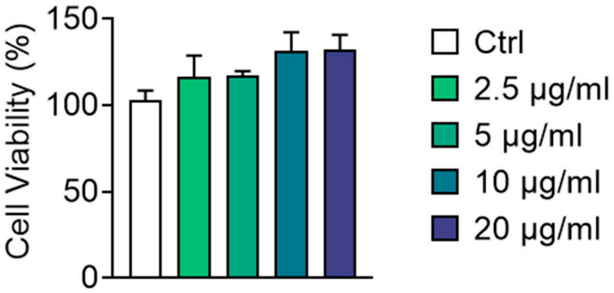

Figure S3: Cell viability of human keratinocytes after limonin treatment. 5×10^4 HaCaT cells were seeded in 96-well plates and treated with various concentrations of limonin for 24 h. Cell viability was assessed using the MTT assay. The data are showed as the mean ± SEM. SEM, standard error of the mean; HaCaT, human keratinocytes.
